# Synthesis and Rheological Characterization of Water-Soluble Glycidyltrimethylammonium-Chitosan

**DOI:** 10.3390/md12115547

**Published:** 2014-11-20

**Authors:** Syang-Peng Rwei, Yu-Ming Chen, Wen-Yan Lin, Whe-Yi Chiang

**Affiliations:** Institute of Organic and Polymeric Materials, National Taipei University of Technology, 1, Sec. 3, Zhongxiao E. Rd., Taipei 10648, Taiwan; E-Mails: t7350304@gmail.com (Y.-M.C.); q121926@gmail.com (W.-Y.L.); wheyichiang@gmail.com (W.-Y.C.)

**Keywords:** chitosan (CS), glycidyltrimethylammonium chitosan (GTMAC-CS), maxwell model, relaxation time, frequency-concentration master curve, sol-gel transition

## Abstract

In this study, chitosan (CS) grafted by glycidyltrimethylammonium chloride (GTMAC) to form GTMAC-CS was synthesized, chemically identified, and rheologically characterized. The Maxwell Model can be applied to closely simulate the dynamic rheological performance of the chitosan and the GTMAC-CS solutions, revealing a single relaxation time pertains to both systems. The crossover point of G′ and Gʺ shifted toward lower frequencies as the CS concentration increased but remained almost constant frequencies as the GTMAC-CS concentration increased, indicating the solubility of GTMAC-CS in water is good enough to diminish influence from the interaction among polymer chains so as to ensure the relaxation time is independent of the concentration. A frequency–concentration superposition master curve of the CS and GTMAC-CS solutions was subsequently proposed and well fitted with the experimental results. Finally, the sol-gel transition of CS is 8.5 weight % (wt %), while that of GTMAC-CS is 20 wt %, reconfirming the excellent water solubility of the latter.

## 1. Introduction

Chitin, as the only source of chitosan (CS) and their derivatives, is the second-most abundant natural polymer next to cellulose. Chitin, as a universal template for biomineralized skeletal structures in a broad variety of invertebrates [1], is industrially produced mostly from seashell materials. Chitin has been seen as one of the most important resources from marine natural products to be applied in bio-related fields. However, it is hard to dissolve in any kind of solvent due to high crystallinity. Chitosan, a deacetylation form of chitin, can dissolve in a low pH solution to form a gel owing to the free amine group easily interacting with acid. CS is therefore firstly used in the field of wastewater treatment. Moreover, CS possesses antimicrobial and biocompatible characteristics which underlie its primary use in medicine, including wound dressing and some other medical related areas [2,3]. For example, chitosan has played a leading role in advanced biomaterial applications, including non-viral vectors for DNA-gene and drug delivery because it is non-toxic, stable, biodegradable, and easily sterilized [4,5]. However, CS is not soluble in pure water that limits the use in some daily life areas such as the cosmetic industry and food industry [6,7]. Accordingly, the solubility of CS in pure water must be improved and such an issue has drawn great attention to many scholars [8,9].

Rwei *et al.* have modified chitosan with “1, 3-propane sultone” to produce a novel sulfonated chitosan (SCS) which has great water-solubility [10]. However, the reacting monomer 1, 3-propane sultone is known as a potent human carcinogen [11]. To remove the unreacted sultone, performing a complete reaction followed by additional purification thus becomes crucial and costly for the SCS preparation. In this study, another water-soluble CS-related compound, GTMAC-CS, of which CS is modified by glycidyltrimethylammonium chloride (GTMAC) in order to gain good aqueous solubility, was prepared.

GTMAC is known to be a widely used chemical for starch modification in food and paper industries. Low-substituted cationic starch is commonly prepared by reacting starch with GTMAC [12]. Regarding its medical application, Giammona [13] has been reported to successfully react poly(asparthylhydrazide) (PAHy) with GTMAC for application in the systemic gene delivery. The biocompatibility of PAHy-GTA derivatives with different degrees of positive charge substitution were found to be neither haemolytic nor cytotoxicity. Moreover, Xiao *et al.* [14] and Lim *et al.* [15] have demonstrated the synthesization of chitosan with GTMAC to form 2-hydroxyl-propyl-3-trimethylammonium chitosan chloride, abbreviated as HTCC in their work and denoted as GTMAC-CS herein, for better water solubility and application as a drug delivery carrier. The GTMAC-CS has been reported thereafter to successfully load Parathyroid Hormone-Related Protein 1–34 [16], BSA [17], and insulin [18]. *In vitro* study showed the protein/GTMAC-CS/TPP nanoparticles demonstrate an initial burst then a slow and continuous release [19].

The synthesized compounds of GTMAC-CS in this work were chemically identified by FTIR and NMR. The viscoelastic characterization of GTMAC-CS under various concentrations at a wide range of frequencies (0.01 to 100 Hz) was performed. The frequency–concentration superposition relationship was built and the sol-gel transition was then investigated. It is our goal to understand the rheological properties of GTMAC-CS aqueous solutions from a dilute state to a gel state and to explore the feasibility of applying it to daily life.

## 2. Experimental Method

Chitosan used in this study was obtained from VA7G Bioscience (Taipei, Taiwan). It possessed a molecular weight (Mn, number average) of about 5 × 10^4^ and had a deacetylation degree (DD) of 90%. A regular CS solution was prepared using deionized water with 5 wt % of acetic acid as a solvent. The acetic acid used herein was purchased from Acros (Pittsburgh, PA, USA) and was used as received.

**Scheme 1:**
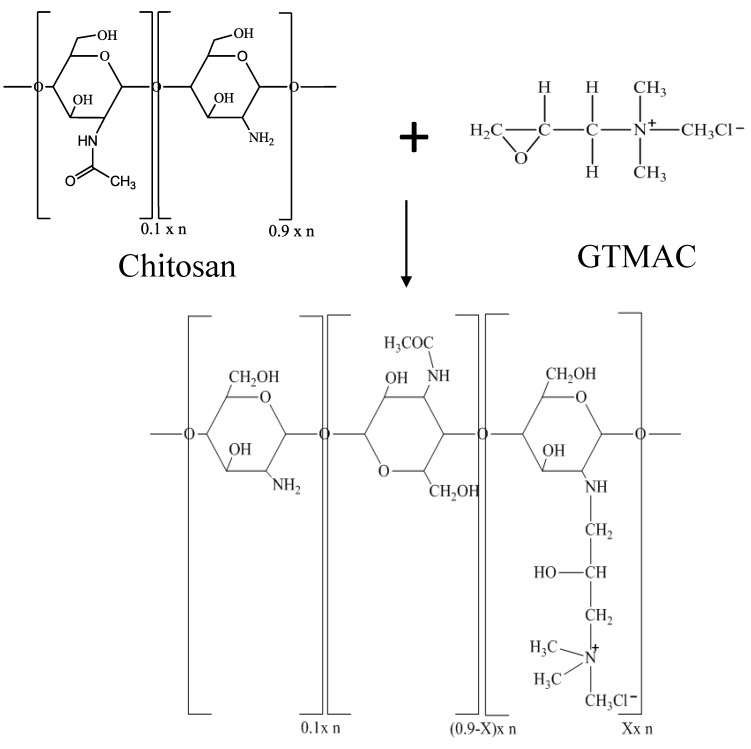
Synthesis procedure of Glycidyltrimethylammonium-chitosan (GTMAC-CS).

The GTMAC-CS was prepared by reacting chitosan with glycidyltrimethylammonium chloride (GTMAC). Five grams of chitosan was completely dissolved in 191 mL of deionized water to which 2 wt % acetic acid was added. In total, 27.6 mL of GTMAC was then injected into the CS solution under a nitrogen environment [11,20]. The reaction proceeded at 50 °C for 18 h. After the reaction, the reacted solution was poured into cold acetone, causing the precipitation of product. The crude solid was washed using methanol to remove the excess glycidyltrimethylammonium chloride. After drying for 6 h in a vacuum oven, the GTMAC-CS was obtained as a white powder at a yield of approximately 80% (Scheme 1).

Infrared spectra were performed on the PerkinElmer Spectrum 100 (PerkinElmer, Waltham, MA, USA). ^1^H NMR spectra were measured using a Bruker Avanceat 500 MHz (Bruker, Santa Barbara, CA, USA). D_2_O was used as a solvent. GPC analyses were carried out using a GPC/V2000 from Waters Co. (Waters, Milford, MA, USA) and AcOH (Acetic acid) was selected as an eluent. The steady viscosity was measured for pure solvent and polymer solution by the rheometer of Brookfield DV-III (Brookfield, Middleboro, MA, USA) plus to obtain the specific viscosity. Dynamic rheology was examined using a strain-controlled rheometer, Vilastic (Vilastic Scientific, Austin, TX, USA), which produced an oscillatory flow using a vibratile membrane, and detected the instantaneous pressure variation as a stress-response. The samples were examined under dynamic shear of constant but low amplitude within the linear viscoelastic range. The frequency was swept over the range 10^−2^ Hz < *ω* <10^2^ Hz, the real part and the imaginary part of the shear moduli, representing the storage modulus G′ and loss modulus Gʺ, respectively, were obtained. A detailed description of the instrument and measurement can be found in the authors’ other work [10,21,22].

## 3. Results and Discussion

**Figure 1:**
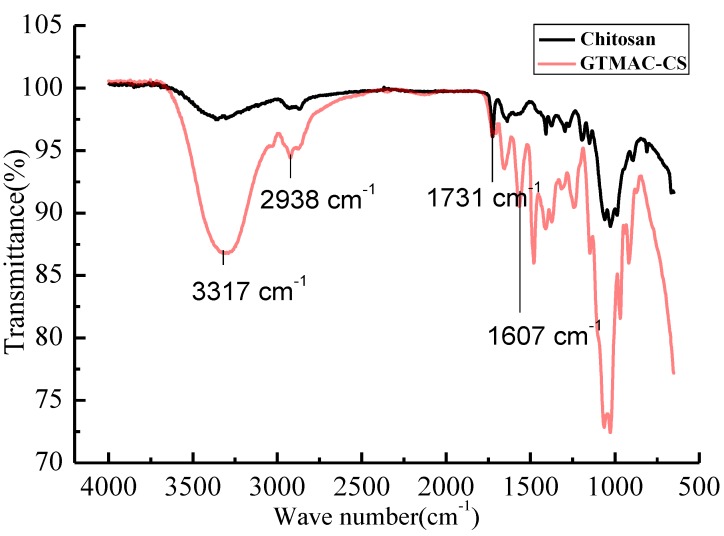
The IR spectra of CS and GTMAC-CS.

The FT-IR spectrum to verify the GTMAC-CS synthesis was shown in Figure 1. The spectrum included strong adsorption at 1607 cm^−1^, corresponding to the C-N-C bending vibration of the GTMAC-CS branch. The C = O stretching vibration of the amide group from the chitin segment, occurring at 1731 cm^−1^, can be considered as an invariant peak which should be the same for both CS and GTMAC-CS polymers. The adsorption centered at 2938 cm^−1^ was attributed to the C-H stretching vibration of the CH_2_ or CH_3_ groups. Finally, a broad peak centered at 3317 cm^−1^ represented the combined O-H stretching vibration and N-H stretching vibration.

**Figure 2:**
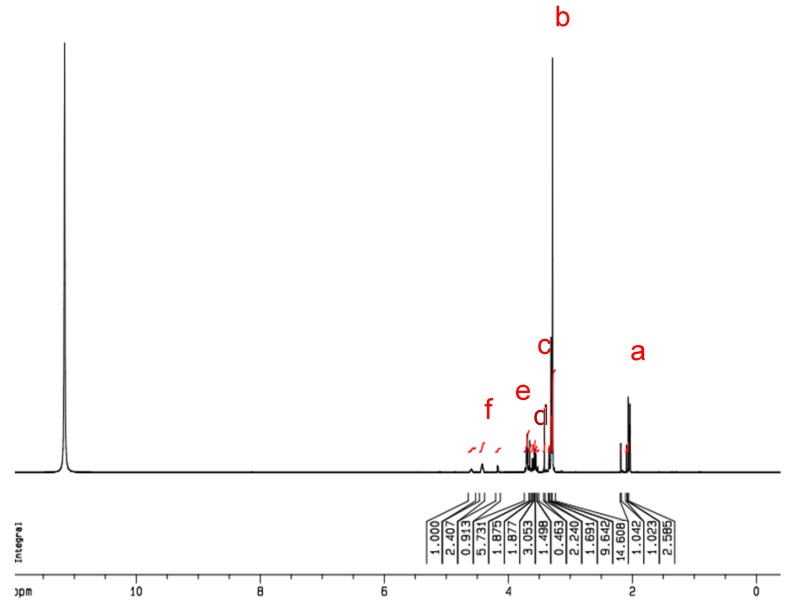
The ^1^H NMR spectra of GTMAC-CS. N(CH_3_)_3_ area/total area = 14.608/51.648 = 9X/(13 * 0.1 + 11 * (0.9 − X) + 25X); X = 63%.

To further identify the synthesized product of GTMAC-CS, the ^1^H NMR spectrum was performed and IS shown in Figure 2. From the obtained NMR spectrum, peaks at δ = 2.02–2.24 ppm (a), δ = 3.71 ppm (c), and δ = 3.98 ppm (d) were assigned to -COCH_3_ (from chitin), -N-CH-, and -NH, respectively. Notably, peaks at δ = 3.41 ppm (b) and δ = 4.42 ppm (e) were found only for the GTMAC-CS, which represent the existence of the groups of N(CH_3_)_3_ and -N-CH_2_-, respectively. Based on the result of NMR analyses shown in Figure 2, the ratio of grafted segments, X, to the total segments of GTMAC-CS is approximately 63 mole %.

**Figure 3:**
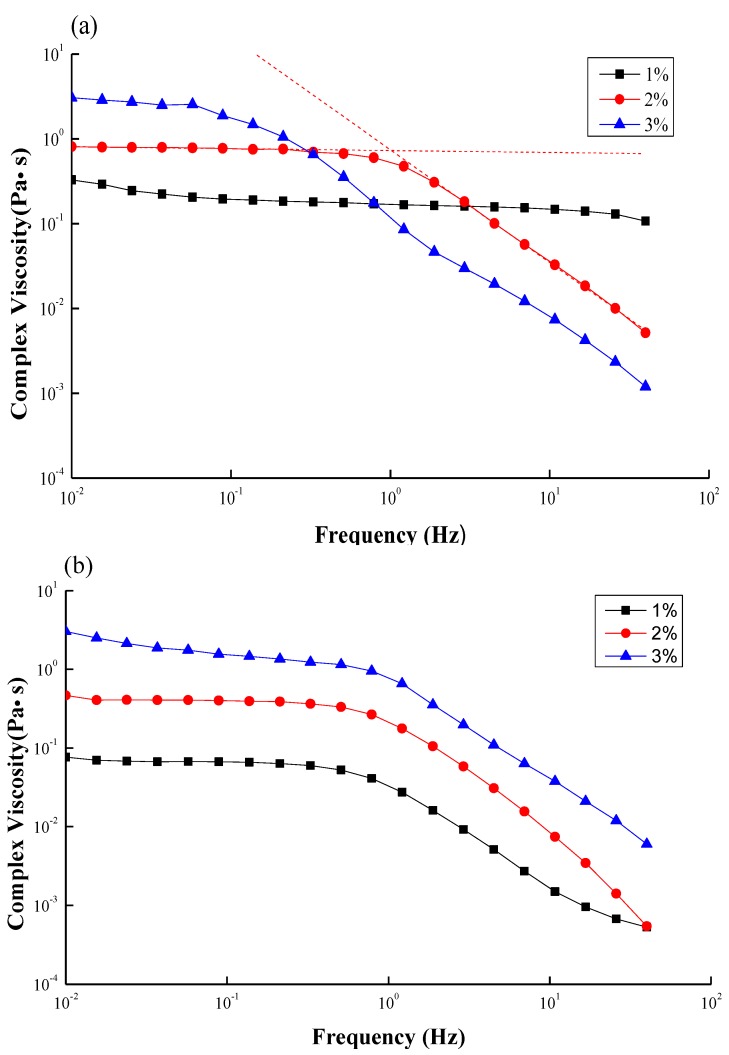
Complex viscosity as function of frequency for (**a**) CS; (**b**) GTMAC-CS solutions at different concentrations (1 wt %~3 wt %).

Figure 3a,b show the complex viscosity as a function of oscillation frequency at different concentrations for CS and GTMAC-CS, respectively. The complex viscosities of both solutions increase with the polymer concentration as usual. Moreover, all the viscosity curves display two regions, a Newtonian plateau region followed by a shear thinning region. The Newtonian behavior indicates that little deformation by the shearing on the chain conformation was created, while the shear thinning phenomena suggests that the chain deformation induced by the stress is too strong to be relaxed and the chain orientation along the flow direction may occur. The inverse of the turning point between the two regions, therefore, can be treated as the relaxation time, λ, representing the longest period of polymer-chains to recover to its original conformation under shearing. A distinguished difference between Figure 3a,b was that the turning points of CS solutions shift to the low frequency region as the concentration increases while those of GTMAC-CS solutions remained the same. In general, a given shearing stress would generate greater torque for a linear polymer with a higher molecular weight, which would thus orientate the polymer easier to yield a turning point at the lower-frequency region. In a highly concentrated environment, the CS polymers might be entangled together to have a bigger conformation and exhibit longer relaxation time. However, good water solubility of GTMAC-CS would cause some lubrication effect to prevent the entanglement. The inverse of the turning point, namely, the relaxation time, therefore was independent of the concentration.

**Figure 4:**
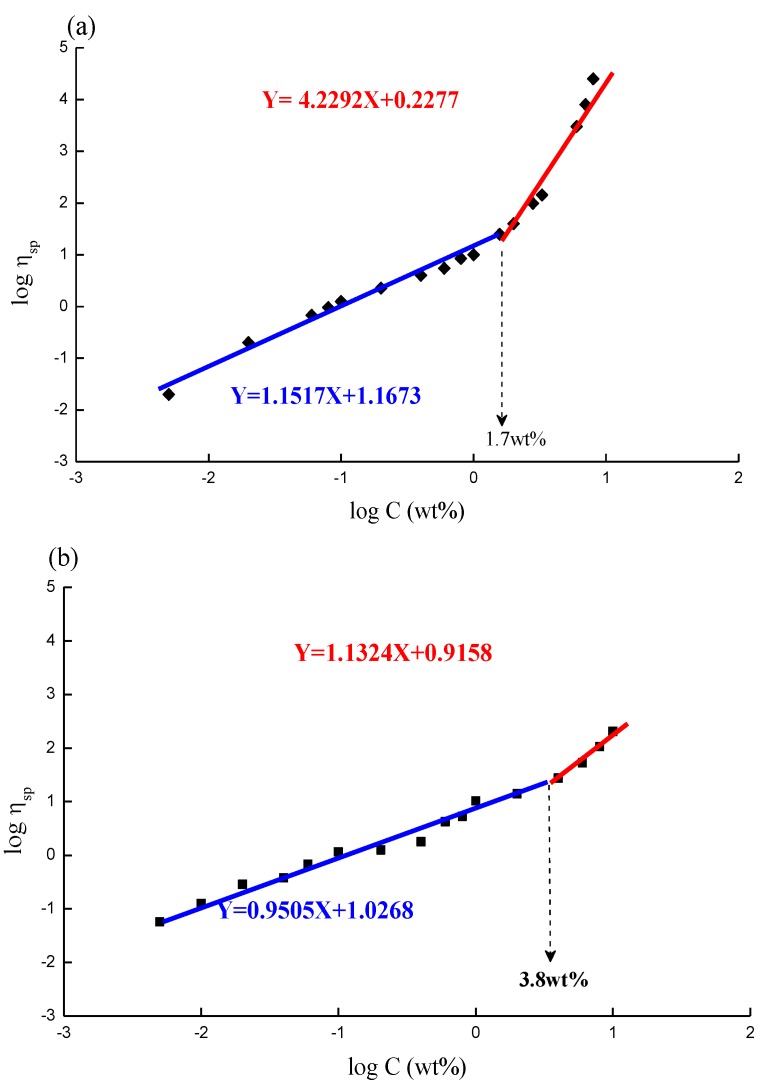
The overlap concentration Ce of (**a**) CS; (**b**) GTMAC-CS solutions.

Figure 4a,b depict the plots of specific viscosity against polymer concentration in a log scale; a critical concentration point, Ce, can be found at 1.7 wt % and 3.8 wt % for CS (Figure 4a) and GTMAC-CS (Figure 4b), respectively. Moreover, the slopes at the concentration range lower and higher than Ce are 1.1 and 4.2, respectively, for CS (Figure 4a) and 0.9 and 1.2, respectively, for GTMAC-CS (Figure 4b). Increasing polymer concentration enhances the interaction among the polymer chains. If the polymer concentration exceeds a threshold value, usually symbolized as Ce, the response of the polymer to the oscillation is no longer merely affected by the individual molecular size of dissolved polymer; it will also depend on the chain–chain interactions in the conformation-overlapped zone, usually called “chain-entanglement” among polymers. When the polymer concentration is increased from a dilute to a concentrated condition over such a critical threshold, Ce, the individual chains have chances to be in contact with each other. Further increasing the polymer concentration (C > Ce), the crowded molecular chains would be entangled and act as if they were in a molten state. For random-coil polymer solutions, the dependence of specific viscosity at zero shear rate on the concentration C therefore yields an increasing trend with concentration, *i.e.*, from a linear to an exponential dependence. The exponent of C usually equals to 1 for C < Ce and 3~5 for C > Ce. The viscosity data presented in Figure 4a,b show a regular behavior of random-coil polymer solutions [23,24,25,26]. Interestingly, Figure 4a,b exhibit CS has lower Ce and higher exponent values at the concentrated region than GTMAC-CS does, indicating the solubility of GTMAC-CS in pure water is much better than that of CS in an aqueous solution mixed with 5% acetic acid. Good water solubility can prevent GTMAC-CS from entanglement because water molecules persistently attaching to polymer chains function as lubricants to prevent polymer chains from entanglement. Such a character can be confirmed by the constant relaxation time of GTMAC-CS with respect to various concentrations investigated in this work.

**Figure 5:**
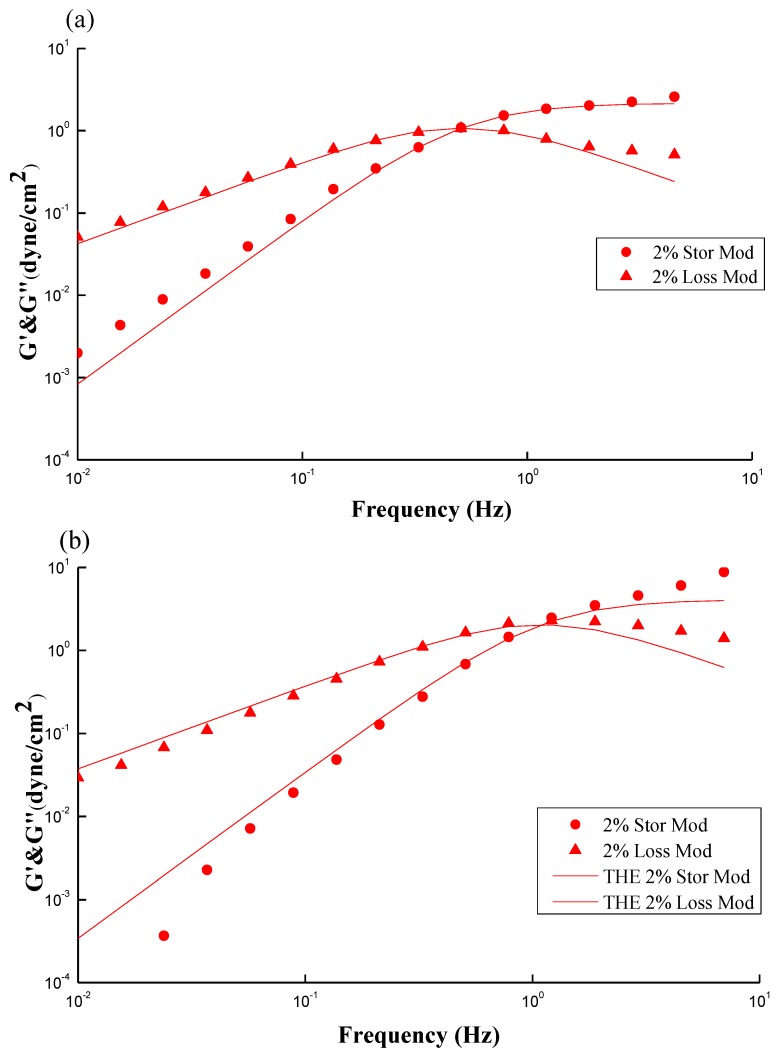
Typical plots of a dynamic rheological measurement on (**a**) 2 wt % CS; and (**b**) 2 wt % GTMAC-CS solutions. The data was simulated by the Maxwell model (solid line).

Figure 5a,b show typical plots of a dynamic rheological measurement on CS and GTMAC-CS solutions, respectively. The storage (G′) and loss (Gʺ) moduli increase with frequency. The storage modulus G′ generally symbolizes the elastic behavior while the loss modulus Gʺ symbolizes the viscous behavior; both of them can be obtained by a small amplitude oscillation shearing test (SAOS) within a linear viscoelastic range. However, as the frequency keeps increasing, Gʺ decreases beyond the crossover point, while G′ increases toward a plateau region. Notably, the inverse of frequency at the cross-over point of G′ and Gʺ can also yield a relaxation time, λ, which reveals the longest time required for the polymer structures in the fluid to relax. As mentioned earlier about the turning point in Figure 3, the results shown from Figure 5 can offer another way to obtain the relaxation time of polymer under a SAOS test [27,28].

A Maxwell model (Equations (1) and (2)), consisting of a dashpot (viscosity element) connecting with a spring (elasticity element) in series, was used to describe the rheological behavior of Figure 5 [29,30,31,32,33].

G′ = G_∞_(λω)^2^/[1 + (λω)^2^] (1)

Gʺ = G_∞_λω/[1 + (λω)^2^] (2)

where ω is the oscillation frequency; G_∞_ represents a fitting factor, roughly equals double the maximum of the Gʺ; and λ denotes the relaxation time as mentioned above. Theoretically, the limiting slopes of log(G′) and log(Gʺ) against log(ω) before the crossover point are 2 and 1, respectively. Figure 5a,b show that the simulated results (solid line) are in good agreement with the experimental data (points) at oscillation frequencies from 0.01 to 1 Hz. The limiting slopes of log(G′) *vs.* log(Gʺ) for CS and GTMAC-CS solutions are 0.9 *vs.* 1.9 and 1.1 *vs.* 2.1, respectively, indicating that both structural buildup (G′) and breakdown (Gʺ) log-linearly increase with frequency in the frequency range less than 1 Hz. In general, a Maxwellian behavior observed within a low frequency range (<10 Hz) as shown in this study indicates that the chain conformation of dissolved polymers is a random-coiled type with a single relaxation time, namely, a good Gaussian type of polymer chains with narrow distribution [34,35,36]. Goodwin further illustrated that a good match of the Maxwell model reveals an insignificant or even complete lack of aggregation by the hydrophobes [37,38]. Dissolved polymers with some hydrophobic nature will increase the size of aggregates, reduce the number of aggregates, and thereby decrease both energy storage and energy loss during oscillation. Accordingly, the G′ and Gʺ are not sensitive to the frequency and their slopes are less than 2 and 1, respectively. Annable *et al.* [27] gave a similar explanation [22]. In short, the strong fit of Maxwell model in Figure 5a,b reveals a single relaxation time and negligible aggregations for low pH CS solution and regular GTMAC-CS aqueous solution.

**Figure 6:**
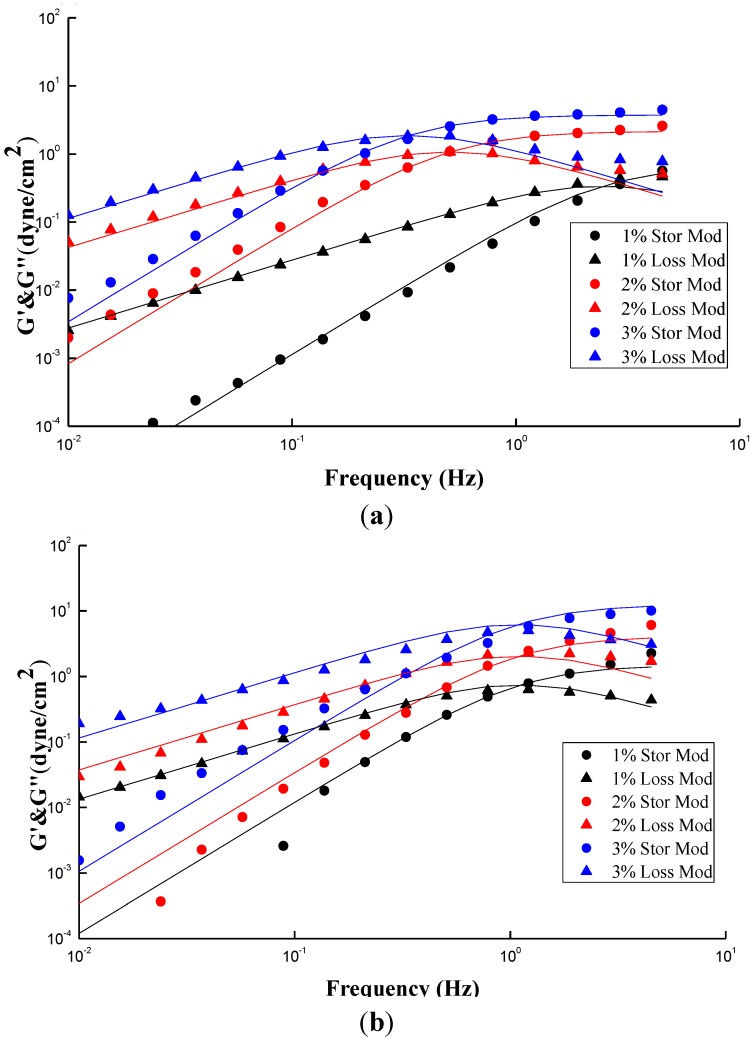
Storage modulus (G′) and loss modulus (Gʺ) for (**a**) CS; (**b**) GTMAC-CS solutions at different concentrations (1 wt %~3 wt %). The data was simulated by the Maxwell model (solid line).

Figure 6a,b show the plots similar to Figure 5a,b except two additional conditions with different concentrations, *i.e.*, 1% and 3%. The fitting parameters and simulation deviations were tabulated in Table 1. An interesting fact similar to Figure 3a, Figure 6a shows an increase of concentration results in an increase in both G′ and Gʺ, and simultaneously a shift in the crossover frequency toward a lower value, revealing an increase of relaxation time with CS concentrations. Notably, the relaxation time of “entangled” CS molecules is increased with concentration once the threshold Ce was passed over, eventhough all of the chain relaxation is still in a well dissolved state with no occurrence of bulky aggregation, and besides the G′ and Gʺ can be described by the Maxwell model. Figure 6a demonstrates that the investigated concentration range of CS solution, 1–3 wt %, is beyond its Ce value of 2 wt %; a longer relaxation time is therefore expected. In contrast, Figure 6b shows that the frequency at which G′ and Gʺ of GTMAC-CS are across from each other is independent of concentration, meaning that the relaxation time is constant. The Ce of GTMAC-CS, obtained from Figure 4b, is 4 wt %, which is far beyond the tested range herein, indicating the existence of the non-entangled condition for the GTMAC aqueous solution. Interestingly, the small deviations of G′ and Gʺ, except the 3 wt % GTMAC-CS condition, shown in Table 1 confirmed the previous statement that the experimental result under SAOS from 0.01 to 1 Hz could be well fitted by a Maxwell model with a single relaxation. Notably, the relatively high deviation of GTMAC-CS 3 wt % might be due to the high intermolecular hydrogen bonds from the hydroxyl groups of GTMAC-CS at a nonacidic environment. The intermolecular hydrogen bonds will prevent the GTMAC-CS polymers from freely random coil behavior which is the basic assumption of applying the Maxwell model.

**Figure 7:**
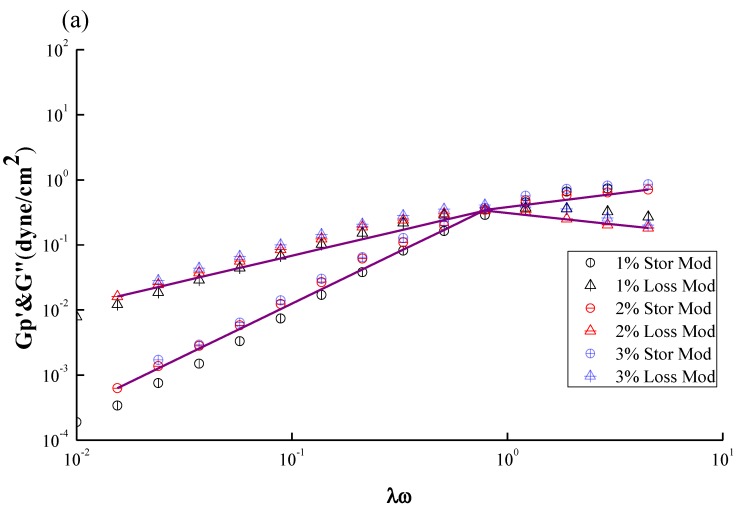

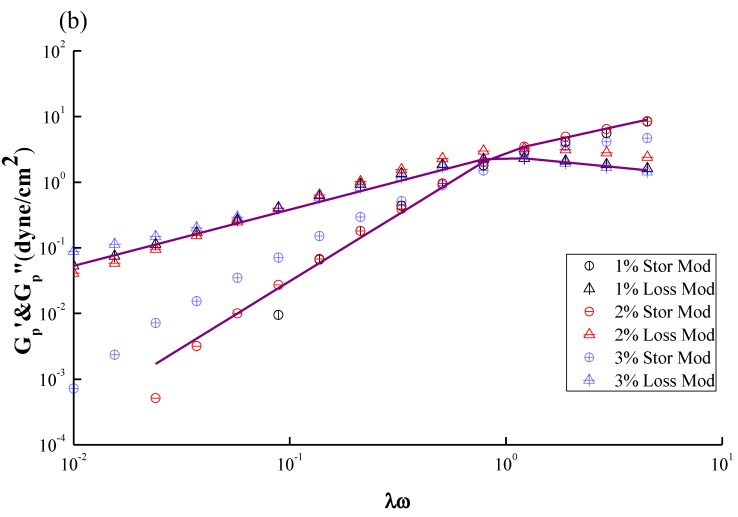
Master curve of frequency–concentration superposition for (**a**) CS; (**b**) GTMAC-CS solutions at different concentrations (1 wt%~3 wt%) shown in Figure 6.

Figure 7a,b show the master curve based on the generalized parameter of G′ or Gʺ against the dimensionless frequency of Figure 6. The idea regarding the concentration shifting was adapted from WLF Equations and can be expressed by Equations (3) and (4).

G′_p_ = G′/a_v_, a_v_ = (C/C_0_), a_h_ = λω (3)

Gʺ_p_ = Gʺ/ a_v_, a_v_ = (C/C_0_), a_h_= λω (4)

where G′_p_ and Gʺ_p_ are defined as the “reduced moduli” which is normalized by using concentration ratio, a_v_, as a vertical shifting factor. The horizontal shifting factor, a_h_ and the vertical shifting factor, a_v_, equal to the dimensionless groups “λω” and “(C/C_0_): the ratio of the given concentration to the target concentration which is 2 wt % in this work”, respectively. Such a superposing concept is inspired by the derivation of the well-known WLF shifting equation [39,40,41] and has been thoroughly discussed in our previous paper. Figure 7a,b show that both GTMAC-CS and CS exhibit good shifting into a single curve of G′ and Gʺ, respectively, revealing the absence of a conformational transition or the formation of a supermolecular structure under the selected operating conditions. However, the shift along the y-axis still shows a certain degree of scattering for both CS and GTMAC-CS solutions. The concentration shift based on Equations (3) and (4) actually assumes a linear relationship with the loss modulus as well as the storage modulus, which lacks some theoretical support and requires further verification in the future.

**Table 1:** The fitting parameters and simulation accuracy of CS and GTMAC-CS solutions determined from Figure 6.

	Concentration	ω_c_ (Hz)	λ(s)	G_∞_(dyne/cm^2^)	Dev.G′ #	Dev.Gʺ #
CS	1 wt%	2.43	0.41	0.31	0.20	0.02
	2 wt%	0.51	1.96	2.20	0.06	0.03
	3 wt%	0.33	3.03	4.52	0.04	0.02
GTMAC-CS	1 wt%	1.02	0.98	0.66	0.02	0.09
	2 wt%	1.09	0.91	2.45	0.04	0.00
	3 wt%	1.08	0.92	6.35	0.10	0.19

#: $Dev.G^{\prime }=\frac{\left|G^{\prime }\exp-G^{\prime }the\right|}{G^{\prime }the}$; $Dev.G^{\prime \prime }=\frac{\left|G^{\prime \prime }\exp-G^{\prime \prime }the\right|}{G^{\prime \prime }the}$, where G_exp_ and G_the_ represent the average value −of G obtained from experiment and fitting data, respectively. The data of G′ or Gʺ within the frequency less than ω_c_ are used to obtain the averaged values.

**Figure 8:**
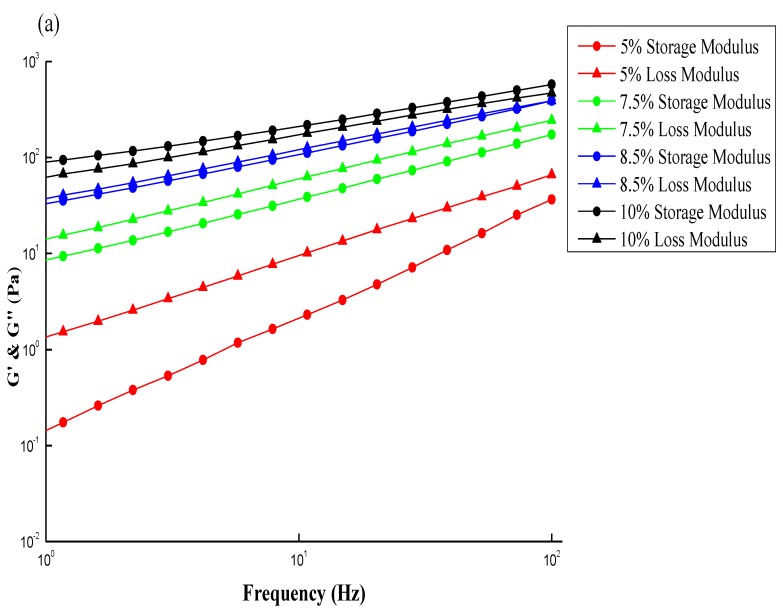

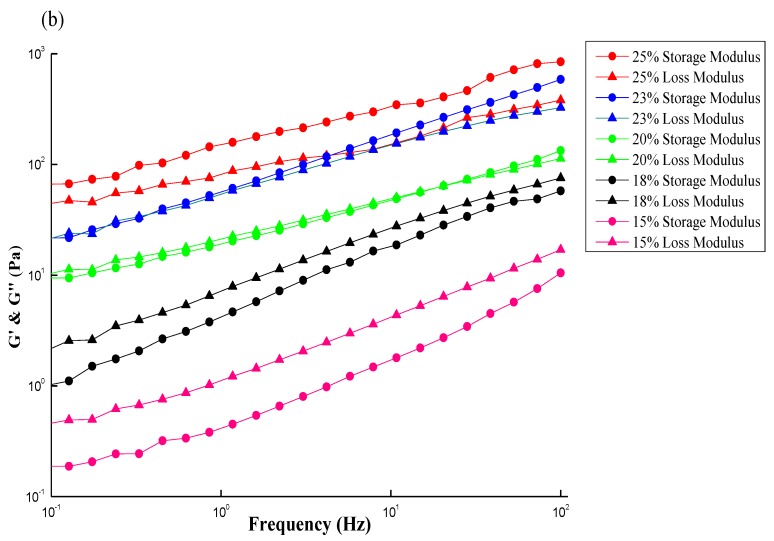
Dynamic moduli (G′, Gʺ) *versus* frequency to obtain the sol–gel transition concentration for (**a**) CS; (**b**) GTMAC-CS solutions.

## 4. Conclusions

Chitosan grafted by GTMAC to form GTMAC-CS was successfully produced and rheologically characterized herein. The Maxwell model can be applied to simulate the dynamic rheological performance of the CS and the GTMAC-CS solutions, revealing a single relaxation time exists for both systems. The crossover point of G′ and Gʺ shifted toward lower frequencies as the CS concentration increased but remained constant as the GTMAC-CS concentration increased, indicating the solubility of GTMAC-CS in water is good enough to eliminate the influence from the interaction among polymer chains so as to make the relaxation time independent of the polymer concentration. A frequency–concentration superposition master curve was subsequently proposed and well fitted with the experimental results for both CS and GTMAC-CS solutions. Finally, the sol–gel transition concentration was investigated, which was 8.5 wt% and 20 wt% for CS and GTMAC-CS, respectively, reconfirming the excellent water solubility of the latter.
